# Resveratrol Reverses Retinoic Acid Resistance of Anaplastic Thyroid Cancer Cells via Demethylating CRABP2 Gene

**DOI:** 10.3389/fendo.2019.00734

**Published:** 2019-10-29

**Authors:** Xin Liu, Hong Li, Mo-Li Wu, Jiao Wu, Yuan Sun, Kai-Li Zhang, Jia Liu

**Affiliations:** ^1^Liaoning Laboratory of Cancer Genetics and Epigenetics, Department of Cell Biology, College of Basic Medical Sciences, Dalian Medical University, Dalian, China; ^2^Research Center, South China University School of Medicine, Guangzhou, China

**Keywords:** retinoic acid, resveratrol, DNA methylation, CRABP2, DNA methyltransferase

## Abstract

**Background:** Cellular retinoic acid binding protein 2 (CRABP2) mediates retinoic acid/RA anti-cancer pathways. Resveratrol effectively reverses RA tolerance and upregulates CRABP2 expression of anaplastic thyroid cancer cell line THJ-11T. As DNA methylation is responsible for CRABP2 silencing, the CRABP2 methylation status of THJ-11T cells and the demethylating effect of resveratrol on this gene are elucidated.

**Materials and methods:** The statuses of CRABP2 expression and methylation and the levels of DNA methyltransferases (DNMTs) DNMT1, DNMT3A, and DNMT3B of THJ-11T cells were examined before and after resveratrol treatment via multiple experimental methods. The human medulloblastoma UW228-2 cell line was cited as the control of CRABP2 methylation and gemcitabine as the demethylator control.

**Results:** RT-PCR, immunocytochemical staining and Western blotting showed that resveratrol significantly increased the CRABP2 expression and RA sensitivity of THJ-11T and UW228-2 cells. Bisulfite sequencing showed five CpG methylation sites at the CRABP2 promoter region of both cell lines, which were partially (3/5) demethylated by resveratrol and totally (5/5) by gemcitabine. DNMT1, DNMT3A, and DNMT3B were reduced in UW228-2 cells and DNMT1 and DNMT3A were reduced in THJ-11T cells after resveratrol treatment in a time-related fashion.

**Conclusion:** Resveratrol is able to erase CRABP2 methylation and can thereby increase the RA sensitivity of THJ-11T and UW228-2 cells. This study demonstrates the additional value of the natural polyphenolic compound resveratrol as a demethylator in cancer treatments.

## Introduction

All-trans retinoic acid (RA) has been commonly used in cancer chemotherapy (1, 2). Its biological effects are largely determined by two classical signaling pathways mediated by Cellular retinoic acid binding protein 2 (CRABP2) or Fatty acid-binding protein 5 (FABP5), which results in tumor suppression or tumor promotion (3). Anaplastic thyroid cancer (ATC) is the most lethal thyroid malignancy; post-diagnosis survival time is <1 year due to its strong invasiveness and frequent metastasis (4, 5). Although ATCs are <2% of thyroid cancer cases, its mortality rate accounts for 33–50% of thyroid cancer-related death (6). RA has been used to enhance the radio-sensitivity of thyroid cancers via promoting thyroid cancer re-differentiation and, therefore, radioactive iodine uptake (7). However, the therapeutic effect of RA is controversial (8). Our recent data demonstrate that RA is ineffective or even promotes ATC cell growth, presumably due to low or absent CRABP2 expression (9), which is suggestive of the necessity to cautiously use RA in anti-ATC therapy. On the other hand, resveratrol effectively suppresses the growth of two RA-tolerant ATC cell lines (THJ-16T and THJ-21T); more interestingly, resveratrol fails to inhibit ATC THJ-11T cell growth by itself but causes extensive cell death when combined with RA (9). Further analysis revealed that resveratrol significantly upregulated CRABP2 expression and reversed the RA tolerance of THJ-11T by opening the RA tumor suppression pathway. These results demonstrate for the first time the therapeutic advantage of resveratrol for ATCs by itself or in combination with RA. Meanwhile, it would be worthwhile to shed light on the underlying mechanism of resveratrol-upregulated CRABP2 expression.

It has been recognized that RA anticancer signaling is activated when RA binds with CRABP2. CRABP2 transports lipophilic RA to the nucleus; RA then passes through heterodimers with its nuclear receptor (RARα/β/γ and RXRα/β/γ) to regulate its target gene expression, leading to differentiation, cell cycle arrest, and apoptosis of the treated cells (10). Therefore, the status of CRABP2 expression is considered a critical element in determining RA sensitivity in cancer cells, including those of medulloblastoma and pancreatic cancers (11, 12). We have found that DNA methylation is responsible for the low or silenced CRABP2 expression in medulloblastoma cells (12, 13). DNMT inhibitor 5-aza-2′-deoxycytidinecytidine (5-aza) is thus able to restore CRABP2 expression and reverse RA resistance of UW228-2 medulloblastoma cells (12). However, 5-aza causes several side effects including gene mutations and chromosomal rearrangements, and it may also affect embryonic development (14, 15), suggesting the necessity to explore lesser toxic demethylation agents such as resveratrol. The finding of recovered CRABP2 expression in resveratrol-treated anaplastic thyroid cancer cells has encouraged us to investigate the possible demethylating capacity of this polyphenol agent.

In order to shed light on the mechanism of resveratrol-upregulated CRABP2 expression and its relevance to RA sensitivities, the suitable cell model(s) would be required. Therefore, ATC THJ-11T and medulloblastoma UW228-2 cell lines were selected for this study because of their absence in CRABP2 expression and tolerance to RA treatment (12). Moreover, UW228-2 cells were found with CRABP2 methylation (12), and the recovery of CRABP2 expression increased their RA sensitivity. This study thus aims to check the methylation status of CRABP2 and the expression patterns of methylation enzymes (DNMT1, DNMT3A, and DNMT3B) in these two cell lines before and after resveratrol treatment and then compared the corresponding parameters obtain from gemcitabine-treated cells.

## Materials and Methods

### Cell Lines and Cell Culture

Anaplastic thyroid cancer ATC (THJ-11T) cell lines were provided by Quentin Liu, Institute of Cancer Stem Cell, Dalian Medical University (16). The medulloblastoma cell lines (UW228-2) were kindly provided by the Department of Neurological Surgery, University of Washington at Seattle (12). The THJ-11T cell line was maintained in RPMI 1640 (GE Healthcare Life Sciences, HyClone Laboratories, Utah, USA) supplemented with 10% fetal bovine serum (FBS, Gibco, Grand Island, NY, USA). The UW228-2 cell line was cultured in DMEM (Invitrogen, Grand Island, NY, USA) containing 10% fetal bovine serum (FBS, Gibco, Grand Island, NY, USA), penicillin (100 U/ml), and streptomycin (100 mg/ml) and was maintained in a humidified incubator at 37°C with 5% CO_2_.

### Cell Treatments

Resveratrol (Res), all-trans retinoic acid (RA; Sigma-Aldrich, St. Louis, MO, USA), and Gemcitabine (GEM; Sigma-Aldrich, St. Louis, MO, USA) were dissolved in dimethylsulfoxide (DMSO, Sigma-Aldrich) to 100, 80, and 50 mM stock concentrations, respectively, and diluted with a culture medium to an optimal working concentration of 100 (16), 10 (12), and 10 μM (17) just before use. THJ-11T and UW228-2 cells were treated with 100 μM resveratrol and 10 μM gemcitabine for 12, 24, 48 h. A total of 5 × 10^4^/ml of the cells were plated to culture dishes (Nunc A/S, Roskilde, Denmark) and incubated for 24 h before further experiments. Dozens of cell-bearing coverslips were concurrently prepared under the same experimental condition using the high throughput coverslip-preparation dishes (Jet Biofile Tech. Inc., Guangzhou, China; China invention patent No. ZL200610047607.8), which were collected during drug treatments, fixed with cold acetone, and used for haematoxylin and eosin (H/E) staining, immunocytochemical labeling (ICC), and TUNEL apoptosis assay. The experimental groups were set in triplicate, and the experiments were repeated three times to establish a definite conclusion.

### Cell Proliferation and Apoptotic Death Assays

Hematoxylin-Eosin (H/E) staining was used to observe the morphological changes of THJ-11T cells treated with resveratrol alone, retinoic acid alone, resveratrol, and retinoic acid. A terminal deoxynucleotide transferase (TdT)–mediated dUTP-biotin nick-end labeling (TUNEL) assay was employed to detect apoptotic cells according to producer's instructions (Promega Corporation, USA). The effect of drug treatment on cell proliferation was determined by 3-[4,5-dimethylthiazol-2-yl]-2,5-diphenyl-tetrazolium bromide (MTT) assay. Cells (1 × 10^4^/well) were plated in 96-well flat-bottomed culture plates (Falcon, Becton–Dickinson Labware, Franklin Lakes, NJ, USA) and routinely cultured in 100 μl RPMI1640 medium for 24 h, followed by 48 h of 100 μM Res, 10 μM RA, or their combination (Res/RA) treatment. By the end of the treatments, MTT reagent was added to each well and the plate was incubated for 4 h at 37°C. DMSO was added and the optical density (OD) of the sample plate was measured at 492 nm in a microplate reader.

### Genomic DNA Extraction and Bisulfite Sequencing PCR

By the method described elsewhere (12), DNA samples were extracted from THJ-11T and UW228-2 cells, placed in a 1.5-ml Eppendorf tube containing 10–30 μl of a 1% sodium dodecyl sulfate-containing TE buffer (Tris.Cl + EDTA, pH8.0). Bisulfite treatment of the isolated DNA was carried out using a BisulFlash DNA Modification Kit (EPIGENTEK, USA), according to manufacturer's protocol. A pair of PCR primers in the sequences of CRABP2-F, 5′-GGGTTTTTGTTTAATTTTTTAATGTT-3′ and CRABP2-R, 5′-CCTCACCAAAATAACCTAAATCAA-3′ was used for sequencing critical CpG sites within an intron of the CRABP2 gene (12). According to the protocol provided with the TaKaRa EpiTaq HS (R110Q, TaKaRa), the DNA samples were converted for bisulfite sequencing PCR (BSP) and the selected promoter regions were amplified thereafter.

### Methylation Sequencing and Analysis

The modified DNA samples were amplified using a TaKaRa PCR amplification Kit (TaKaRa Biotech. Inc., Dalian, China) and the products were sequenced in reverse direction by TaKaRa Biotech Inc. using an ABI PRISM^TM^ (Applied Biosystems Inc., Foster City, CA, USA) 3730XL DNA Analyzer and ABI PRISM^TM^ 377XL DNA sequencer. Simultaneously, the sample DNA isolated from UW228-2 cells expressing CRABP2 was used as positive control for promoter methylation. The BSP-generated sequences were compared with the sequence of the corresponding region in the normal human genome (https://www.ncbi.nlm.nih.gov/nuccore/NC_000001.11?from=156699606&to=156706251&report=genbank&strand=true).

### RNA Isolation and RT-PCR

After resveratrol and gemcitabine treatments for 12, 24, and 48 h, total RNA samples were isolated from the cells using Trizol solution (Life Tech, Texas, USA) and subjected to RT-PCR, using the primers specific for CRABP2, DNMT1, DNMT3A, and DNMT3B (Table 1), according to the producer's protocols of Takara RNA PCR kit (AMV) version 3.1 (Takara, Dalian Branch, Dalian, China). The PCR products were separated on 1.2% agarose gel containing ethidium bromide (0.5 mg/ml), visualized, and photographed using the UVP Biospectrum Imaging System (UVP Inc., Upland, CA). mRNA levels were normalized to levels of β-actin.

**Table 1:** Primer sequences for conventional RT-PCR and methylation specific PCR.

**Gene**	**Primers**	**Amplicon size**	**Annealing**
CRABP2	F:5′-ATGCCCAACTTCTCTGGCAA-3′	375 bp	59°C
	R:5′-CGTCATGGTCAGGATCAGTT-3′		
DNMT1	F: 5′-ACC GCT TCT ACTTCCTCGAGGCCTA-3′	335 bp	55°C
	R:5′-GTTGCAGTCCTCTGTGAACACTGTGG-3′		
DNMT3A	F:5′-GGGGACGTCCGcAGcGTCACAC-3′,	280 bp	65°C
	R:5′-CAGGGTTGGACTCGAGAAATCGC-3′;		
DNMT3B	F:5′ -CCT GCT GAATTACTCACGCCCC-3′,	421 bp	65°C
	R:5′-GTCTGTGTAGTGCACAGGAAAGCC-3′;		
ß-actin	F:5′-GCATGGAGTCCTGTGGCAT-3′,	326 bp	58°C
	R:5′-CATGAAGCATTTGCGGTGG-3′		
Primers for bisulfite sequencing PCR			
CRABP2	F:5′-GGGTTTTTGTTTAATTTTTTAATGTT-3′,	215 bp	58°C
	R:5′-CCTCACCAAAATAACCTAAATCAA-3′		

### Protein Preparation and Western Blotting

Proteins were extracted from the drug-treated cells under different experimental conditions. Equivalent amounts of sample proteins were separated by 8–12% SDS-PAGE electrophoresis and transferred to a polyvinylidene difluoride membrane (Amersham, Buckinghamshire, UK). The membrane was blocked with 5% non-fat dry milk (Sigma-Aldrich) in TBS-T (10 mM Tris-HCl, PH8.0, 150 mM NaCl, and 0.5% Tween 20) for 2 h at room temperature and incubated with anti-caspases-3 (Abcam Inc., Cambridge, UK;1:500), anti-CRABP2 (Proteintech, Chicago, IL, USA; 1:200), anti-DNMT1 (Bioss. Inc., Beijing, China; 1:300), anti-DNMT3A (Bioss. Inc., Beijing, China; 1:1,000), and anti-DNMT3B (Bioss. Inc., Beijing, China; 1:300) primary antibodies overnight at 4°C. After three washes with TBS-T, the membrane was incubated for 1.5 h with HRP-conjugated anti-mouse or anti-rabbit IgG (Zymed Lab, Inc.). Protein expression was detected using chemiluminescent ECL reagents (Roche GmbH, Mannheim, Germany). A Gel-Pro analyzer was used to measure the density of bands. Simultaneously, the expression of a target protein was normalized to that of β-Actin.

### Immunocytochemical Staining

Immunocytochemical staining (ICC) was performed on the cell-bearing coverslips obtained from each of the experimental groups by the method described elsewhere (18). The antibodies used were: DNMT1 (Bioss Inc., Beijing, China; 1:400), DNMT3A (Bioss. Inc., Beijing, China; 1:400), DNMT3B (Bioss. Inc., Beijing, China; 1:400), and CRABP2 (Proteintech, Chicago, IL, USA; 1:150). The color reaction was performed by using 3,30-diaminobenzidine tetrahydrochloride (DAB). According to the labeling intensity, the staining results were evaluated by two independent researchers and scored as negative (–) if no immunolabeling was observed in target cells, weakly positive (+) if the labeling was faint, moderately positive (++), and strongly positive (> ++) when the labeling was stronger or distinctly stronger than (++).

### Statistical Analyses

Each experiment was conducted three times, and the data obtained were analyzed together. The results of the MTT cell proliferation assay and cell counting were evaluated with ANOVA and the independent-samples *t*-test. The bar graphs present the mean ± standard deviation (SD) of separate experiments. When required, *p*-values are provided in the figures and their legends.

## Results

### Resveratrol Reversed RA Tolerance

**Figure 1:**
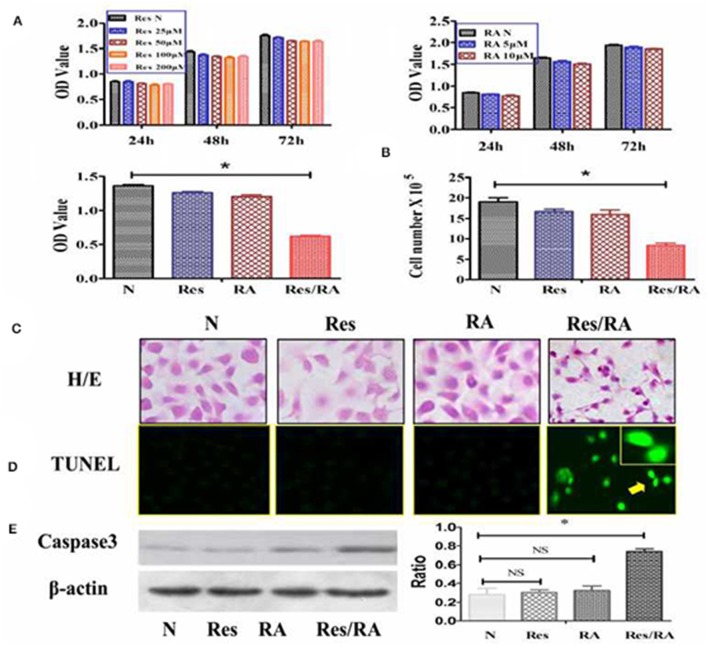
Resveratrol improved RA sensitivity of anaplastic thyroid cancer THJ-11T cells. **(A)** 3-[4,5-Dimethylthiazol-2-yl]-2,5-diphenyl-tetrazolium bromide (MTT) cell proliferation assay; **(B)** viable cell counting after drug treatment for 48 h; **(C)** H&E morphological staining(×40); **(D)** deoxynucleotidyl transferase-mediated dUTP-biotin nick and labeling assay (TUNEL) for apoptotic cell labeling (Green in color; ×40); **(E)** Western Blotting; N, cultured in 0.2% dimethylsulfoxide (DMSO)-containing medium; Res, 100 μM resveratrol treatment; RA, 10 μM retinoic acid treatment; Res/RA, treated with a combination of 100 μM resveratrol and 10 μM retinoic acid for 48 h. Ratio, the ratio between the levels of the target molecules and that of β-actin; NS, no statistical significance (*p* > 0.05); *p < 0.01; the error bars, the mean ± standard deviation. Arrows indicate the region with higher magnification (×80) in the insets.

The MTT cell proliferation assay was conducted on THJ-11T cells treated with Res in the concentrations of 25, 50, 100, or 200 μM and with RA in the concentrations of 5 or 10 μM for 24, 48, and 72 h respectively. As shown in Figure 1A, all of the experimental groups had similar OD values in comparison with that of the 0.2% dimethyl sulfoxide (DMSO)-treated cells (N) (*p* > 0.05). In contrast, the OD value of the 100 μM Res/10 μM RA-treated THJ-11T cells was significantly reduced in comparison with those of other groups (*P* < 0.01). The total number of THJ-11T cells was remarkably decreased (Figure 1B) after 48 h 100 μM resveratrol/10 μM RA treatment (*p* < 0.05). No significant phenotypic change was observed either in the 100 μM Res or in the 10 μM RA treated population, whereas the size of Res/RA-treated cells became smaller with elongated protrusion (Figure 1C). TUNEL assay showed distinct cell death only in the THJ-11T cell population treated by Res/RA combination for 48 h (Figure 1D). The gray density analyses of the Western blotting results showed a 2.6-fold increase of caspase 3 production in Res/RA-treated cells but not in ones treated by Res or RA alone (Figure 1E).

### Resveratrol Upregulated CRABP2 Expression

**Figure 2:**
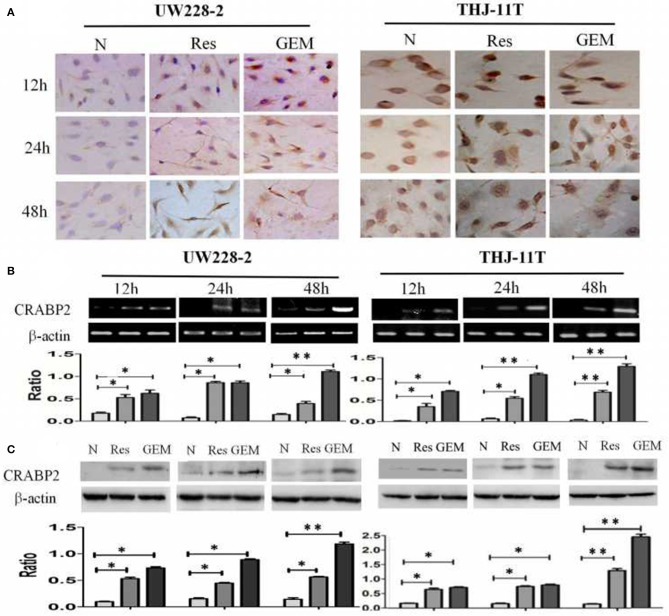
Demonstration of upregulated CRABP2 expression in 100 μM resveratrol (Res) or 10 μM gemcitabine (GEM)-treated THJ-11T and UW228-2 cells. **(A)** Immunocytochemical staining (×40); **(B)** RT-PCR; **(C)** Western blotting. β-actin was used as qualitative and quantitative control. N, cultured in 0.2% dimethylsulfoxide (DMSO)-containing medium; Res, 100 μM resveratrol; GEM, 10 μM gemcitabine. Ratio, the ratio between the levels of the target molecules and that of β-actin; NS, no statistical significance (*p* > 0.05); *with statistical significance (*p* < 0.01; **p < 0.001) the error bars, the mean ± standard deviation.

THJ-11T and UW228-2 cells were treated with resveratrol and gemcitabine for 12, 24, and 48 h, respectively to evaluate the levels of CRABP2 expression. Accompanied by morphological changes, both cell lines showed CRABP2 upregulation by either resveratrol or gemcitabin in a time-related fashion (Figures 2A–C; Table 2). It was also found that CRABP2 levels in gemcitabine-treated THJ-11T and UW228-2 cells were 36 and 33% higher than that of their resveratrol-treated counterparts.

**Table 2:** CRABP2 immunocytochemical staining patterns in UW228-2 and THJ-11T cells under different experimental conditions.

	**12 h**			**24 h**			**48 h**		
	**N**	**Res**	**GEM**	**N**	**Res**	**GEM**	**N**	**Res**	**GEM**
UW228-2	-	+	+	-	+	+	-	++	+++
THJ-11T	-	+	+	-	+	+	-	+	++

### CRABP2 Promoter Methylation in UW228-2 and THJ-11T Cells

**Figure 3:**
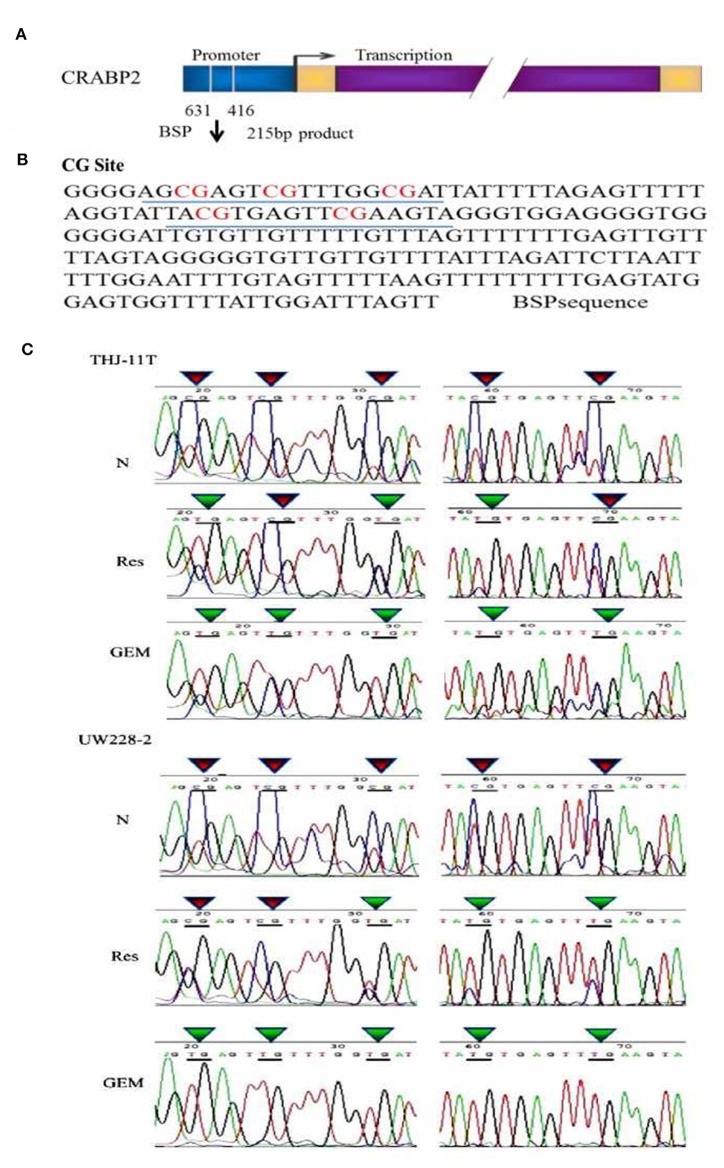
BSP sequencing demonstration of 5 CG site methylation of −631 to −416 CRABP2 promoter region and differential demethylation effects of resveratrol and gemcitabine **(A)** Scheme of CRABP2 gene and the location of BSP amplified promoter region; **(B)** the nucleotide sequence of BSP product and the locations of 5 CG sites; **(C)** demonstration of the sequencing result of the underlined region **(B)** of CRABP2 BSP product generated from UW228-2 and THJ-11T sample DNAs without (N) and with 48 h 100 μM resveratrol (Res) or 10 μM gemcitabine (GEM) treatment. In bisulfite sequencing PCR (BSP), the unmethylated C-bases become U-base in sulfite-treated DNA and are replaced by T-base after PCR; the methylated C-base cannot be converted by sulfite and remain as a C-base in the BSP product. Our sequencing results revealed five CpG methylation sites in the CRABP2 promoter region which were partially (3/5) erased (CpG to TpG) by resveratrol and completely (5/5) erased by gemcitabine.

In accordance with our previous finding (12), the methylation of the CRABP2 promoter region in UW228-2 cells was re-evidenced and therefore cited as the positive control in the sequencing analysis of the CRABP2 PCR product of THJ-11T sample DNA. As shown in Figure 3, five CpG islands in the CRABP2 promoter region of ATC THJ-11T cells and medulloblastoma UW228-2 cells were methylated.

### Partial Demethylation of CRABP2 by Resveratrol in Bisulfite Sequencing

To investigate the effect of resveratrol on methylation of the CRABP2 promoter region, THJ-11T and UW228-2 cells were treated with resveratrol and gemcitabine for 48 h and their CRABP2 PCR products were sequenced. As shown in Figure 3C, the five methylation sites of THJ-11T and UW228-2 were partly (3/5) erased after resveratrol and totally (5/5) after gemcitabine treatment.

### Resveratrol Inhibited DNA Methyltransferase Expression

**Figure 4:**
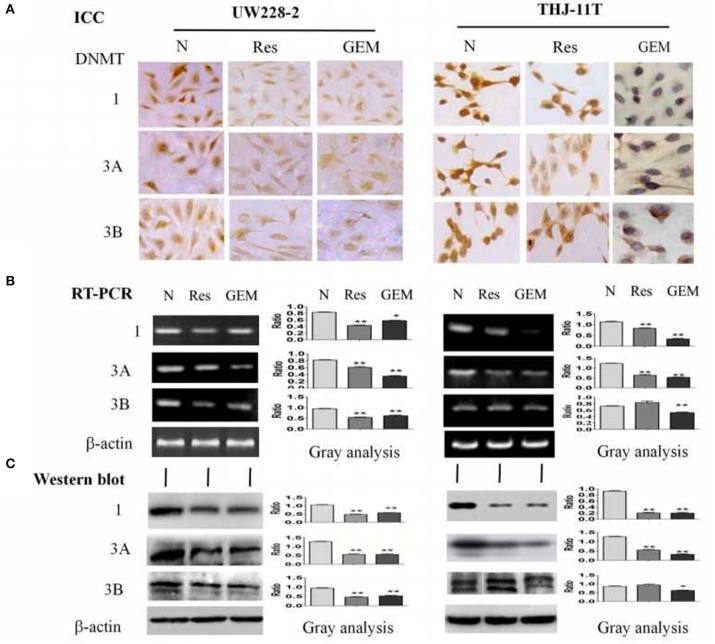
Demonstration of suppressed DNMT1, DNMT3A, and DNMT3B expression in 100 μM resveratrol (Res) or 10 μM gemcitabine (GEM) treated THJ-11T and UW228-2 cells for 48 h. **(A)** Immunocytochemical staining (×40); **(B)** RT-PCR; **(C)** Western blotting. β-actin was used as qualitative and quantitative control. N, cultured in 0.2% dimethylsulfoxide (DMSO)-containing medium; Res, 100 μM resveratrol; GEM, 10 μM gemcitabine. Ratio, the ratio between the levels of the target molecules and that of β-actin; NS, no statistical significance (*p* > 0.05); *with statistical significance (*p* < 0.05); **p < 0.01; ***p < 0.001; the error bars, the mean ± standard deviation.

To disclose the underlying mechanism of resveratrol-mediated demethylation, THJ-11T and UW228-2 cells were treated by resveratrol or gemcitabine for 48 h, followed by RT-PCR, ICC and Western blot examinations for DNA methyltransferase DNMT1, DNMT3A, and DNMT3B, respectively. As shown in Figure 4 and Table 3, the three enzymes in resveratrol-treated UW228-2 cells were obviously downregulated in both RNA and protein levels. DNMT1 and DNMT3A were downregulated in resveratrol-treated THJ-11T cells, while DNMT3B did not change significantly. The expression of three methyltransferases was downregulated in both cell lines after gemcitabine treatment, and their overall reductions were more distinct in comparison with that of resveratrol-treated UW228-2 and, especially, THJ-11T cells.

**Table 3:** DNMT1, DNMT3A, and DNMT3B immunocytochemical staining patterns of UW228-2 and THJ-11T cells cultured for 48 h under different experimental conditions.

	**UW228-2**			**THJ-11T**		
	**N**	**Res**	**GEM**	**N**	**Res**	**GEM**
DNMT1	++	+	+	+++	++	-
DNMT3A	++	+	-	+++	+	-
DNMT3B	++	+	-	+	++	-

## Discussion

CRABP2 has been known to be a central player in RA anticancer signal transduction (19, 20). However, this gene is often down-regulated or silenced by methylation and, therefore, results in RA tolerance in a variety of cancer cells (11–13, 21, 22). For instance, CRABP2 is silenced in the RA-resistant medulloblastoma UW228-2 cells due to CpG island methylation in the promoter region (12). When CRABP2 expression was restored by 5-aza-2′-deoxycytidine, UW228-2 cells showed increased RA sensitivity in terms of growth suppression and apoptosis (10), suggesting that the methylation-silenced CRABP2 expression is responsible for RA tolerance of UW228-2 cells. Our recent data demonstrated that resveratrol could effectively overcome RA resistance of THJ-11T cells in which CRABP2 expression was downregulated (9). Considering the important role of CRABP2 in the RA anticancer pathway, we speculated that resveratrol might enhance the RA sensitivity of THJ-11T via altering the methylation status of CRABP2.

Bisulfite sequencing PCR (BSP) product sequencing has been commonly used to identify gene methylation, because the unmethylated C bases—rather than the methylated C—are changed to U in the sulfite-treated DNA, and only the unmethylated C base is replaced by T after PCR amplification, which can be detected by BSP sequencing (23–25). To ascertain the relevance of resveratrol-upregulated CRABP2 expression with DNA demethylation, the sample DNAs were isolated from medulloblastoma UW228-2 and anaplastic thyroid cancer THJ-11T cells before and after resveratrol treatment and subjected to BSP PCR and the sequencing of BSP products. The results showed that, in a similar fashion to the situation of UW228-2 cells, five methylated CpG sites at the CRABP2 promoter region of THJ-11T cells were methylated. In the case of DNA samples isolated from UW228-2 and THJ-11T cells treated by resveratrol and gemcitabine, CpG island methylation in the CRABP2 promoter region was partly erased (3/5) by resveratrol and totally (5/5) by gemcitabine, indicating that gemcitabine posed a more powerful demethylation effect than resveratrol. Despite this, the results of the current study once again demonstrate the demethylation activity of resveratrol in cancer cells (26). Because DNA methylation is the major cause of drug tolerance in many cancer cells (27–30), gemcitabine and other demethylators are increasingly used in clinical cancer treatments in a single dose or a more comprehensive manner (31–33). Nevertheless, these agents cause some toxic effects, such as gene mutation, chromosomal rearrangement, and embryonic development defects (15). Unlike conventional demethylating agents, resveratrol exerts little adverse effects on normal tissues/cells in anticancer doses (34). Taking CRABP2 as an example, although the demethylation efficacy of resveratrol is not as strong as gemcitabine, it is still sufficient to upregulate CRABP2 expression and consequently reverse RA resistance of UW228-2 and THJ-11T cells. These findings thus provide an alternative approach to treat cancers using resveratrol as an epigenetic regulator.

It has been recognized that DNA methylation is mediated by the DNA methyltransferase (DNMT) enzyme family, of which DNMT1, DNMT3A, and DNMT3B play active roles (35, 36). The DNMTs can catalyze the transfer of methyl group from S-adenosylmethionine to C in CpG dinucleotide and methylation to 5-methylcytosine (5mC) (37). Therefore, the levels of DNA methyltransferase expression are positively correlated with the extent of CpG island methylation (38). In the stepwise carcinogenesis, over-expressed DNMTs lead to methylation silencing of some tumor suppressor genes (39), and downregulation of DNMT expression can reverse the silencing of those genes by weakening their promoter methylation (40–42). Therefore, demethylation of tumor suppressor genes has become an anticancer strategy and suppression of DNA methyltransferase activity with DNMT inhibitors such as gemcitabine is proved as an efficient way (43, 44). To investigate the underlying mechanism of resveratrol-caused demethylation, the patterns of DNMT1, DNMT3A, and DNMT3B expression in UW228-2 and THJ-11T cells before and after resveratrol treatment were analyzed. The results revealed the downregulated DNMT1, DNMT3A, and DNMT3B expression in resveratrol-treated UW228-2 cells and downregulated DNMT1 and DNMT3A in THJ-11T cells. In a similar vein as with the different demethylation efficacies of resveratrol and gemcitabine on CRABP2, gemcitabine shows better suppressive effects on the three DNMTs. Given the above evidence, it would be possible that suppression of DNMT expression may be responsible for resveratrol-caused CRABP2 demethylation, although the causality of these two molecular events remains to be confirmed. Alternatively, this correlative analysis indicates that resveratrol may exert demethylation in the same way as gemcitabine. Given the evidence of the non-toxic and multiple targeting properties of resveratrol, this polyphenol compound may have distinct advantages over gemcitabine and would be an alternative demethylating drug for cancer prevention and treatment. As our *in vitro* results are obtained from the cancer cells treated by a high concentration (100 μM) of resveratrol, the practical anti-ATC values of resveratrol should be further investigated in the animal cancer models by optimizing the dose and the way of resveratrol administration.

Taken together, CpG island methylation in the CRABP2 promoter region is evidenced in RA-resistant human ATC THJ-11T and medulloblastoma UW228-2 cells, which can be largely erased by resveratrol in the same manner as gemcitabine, demonstrating the ability of resveratrol in DNA demethylation. Reduction of DNMT1, DNMT3A, and DNMT3B expression is found in both resveratrol- and gemcitabine-treated cells, which is correlated to the recovered levels of CRABP2 expression. Although the efficacy of the epigenetic regulation of resveratrol is not as powerful as that of gemcitabine, it is still able to resume CRABP2 expression and reverses RA-resistance of the two checked cell lines. In this context, resveratrol can be regarded as either a DNA demethylator or multiple targeting agent in cancer management (45–48). The preventive potential of oral and intraperitoneal administered resveratrol in DEN/MNU/DHPN-induced rat thyroid tumorigenesis may further support this notion (49).

## Data Availability Statement

The raw data supporting the conclusions of this manuscript will be made available by the authors, without undue reservation, to any qualified researcher.

## Author Contributions

K-LZ and JL contributed conception and design of the study. HL organized the database. XL, HL, JW, and M-LW performed the statistical analysis. XL and HL wrote the first draft of the manuscript. XL, HL, and YS wrote sections of the manuscript. All authors contributed to manuscript revision, read, and approved the submitted version.

### Conflict of Interest

The authors declare that the research was conducted in the absence of any commercial or financial relationships that could be construed as a potential conflict of interest.
